# Next generation sequencing predicting clinical effect of immunotherapy on adult rhabdomyosarcoma patient: A case report

**DOI:** 10.1097/MD.0000000000033858

**Published:** 2023-05-26

**Authors:** Xiaogang Sun, Xinyu Wang, Jun Zhou, Yali Xu, Hao Zhang, Mian Xu, Jiaojiao Shen, Xiaoliang Shi, Wei Song, Jun Li

**Affiliations:** a Department of Oncology, Shandong Provincial Hospital Affiliated to Shandong First Medical University, Shandong, China; b Department of Pathology, Shandong Provincial Hospital Affiliated to Shandong First Medical University, Shandong, China; c Shanghai OrigiMed Co., Ltd, Shanghai, China.

**Keywords:** biomarkers, case report, immunotherapy, microsatellite instability, programmed death-ligand 1, rhabdomyosarcoma, tumor mutational burden

## Abstract

Rhabdomyosarcoma (RMS) is a rare sarcoma that rarely occurs in adults and accounts for only 1% of all adult tumors. The standard treatment for RMS is surgical resection, radiotherapy, and chemotherapy.

**Patient concerns:**

Adult patients often present with an aggressive course and poor prognosis.

**Diagnoses:**

The patient was diagnosed with RMS in September 2019 and was confirmed by hematoxylin-eosin staining and immunohistochemistry after surgical resection.

**Interventions:**

The patient received surgical resection in September 2019. He was admitted to another hospital after the first recurrence in November 2019. After the second routine surgical resection, the patient underwent chemotherapy, radiotherapy, and anlotinib maintenance treatment. He relapsed again in October 2020 and was admitted to our hospital. Next-generation sequencing was performed on the punctured tissue of the patient’s lung metastatic lesion, and high tumor mutational burden (TMB-H), high microsatellite instability (MSI-H), and positive programmed death-ligand 1 (PD-L1) were observed. The patient then received toripalimab and anlotinib combined therapy and was evaluated for a partial response after 2 months.

**Outcomes:**

This benefit has persisted for more than 17 months.

**Lessons:**

This is the longest progression-free survival for PD-1 inhibitors in RMS, and there is a trend of continued extension of progression-free survival in this patient. This case supports the hypothesis that positive PD-L1, TMB-H, and MSI-H could be beneficial biomarkers for immunotherapy in adult RMS.

## 1. Introduction

Rhabdomyosarcoma (RMS) is a rare sarcoma affecting children and adults and accounts for 2% to 5% of soft tissue sarcomas (STS). RMS represents a heterogeneous group of cancers with many histological subtypes, and is a malignant neoplasm that normally differentiates to form striated muscles. There are 3 major subtypes of RMS: alveolar, embryonal, and pleomorphic.^[1]^ RMS is the most common type of childhood soft tissue sarcoma, constituting 5% to 10% of all solid tumors in children. However, RMS rarely occurs in adults and accounts for <1% of all adult cancers.

Standard treatments for RMS include chemotherapy (vincristine, actinomycin D, and cyclophosphamide/ifosfamide), radiation therapy, and surgical tumor excision. Although most patients with localized RMS can be cured, the outcomes for those with metastatic or recurrent RMS remain poor. Approximately 23% of RMS patients developed metastasis at diagnosis and long-term event-free survival in patients with metastatic RMS is <20%.^[2,3]^ Therefore, there is a need to develop systemic treatments with better oncological and functional outcomes that consider the long-term safety of patients with RMS. Numerous clinical studies have been performed regarding various treatment strategies, including modified or novel chemotherapy protocols, molecular targeted drug therapies, immunotherapies, and new therapeutic approaches. RMS patients could also benefit from molecularly targeted and immunotherapeutic approaches that reduce treatment-associated toxicities caused by chemotherapy and radiation therapy.

Herein, we report the case of a patient with RMS. Genetic testing revealed tumor mutational burden-high (TMB-H) and microsatellite instability-high (MSI-H), and immunohistochemistry (IHC) showed high programmed death-ligand 1 (PD-L1) expression. The patient was found to have benefited significantly from immune checkpoint blockade treatment.

## 2. Case presentation

A 52-year-old man was admitted to our hospital in May 2019 with a mass on the right side of his back. In May 2018, the patient presented with a soft “quail egg” sized mass on his back that was described as “painless” and ignored. Later, pain began to appear and the mass became significantly larger than in the past. After admission, an magnetic resonance imaging scan demonstrated a paraspinal myometrial lesion within the right portion of his back. Following medical examinations in September 2019, an extended resection of the mass was performed. IHC revealed that the tumor cells were positive for desmin, S100, and myogenin, and negative for CD34, SMA, P63, and CK. Ki-16 index was approximately 50%, which is consistent with the diagnosis of rhabdomyosarcoma.

**Figure 1.**
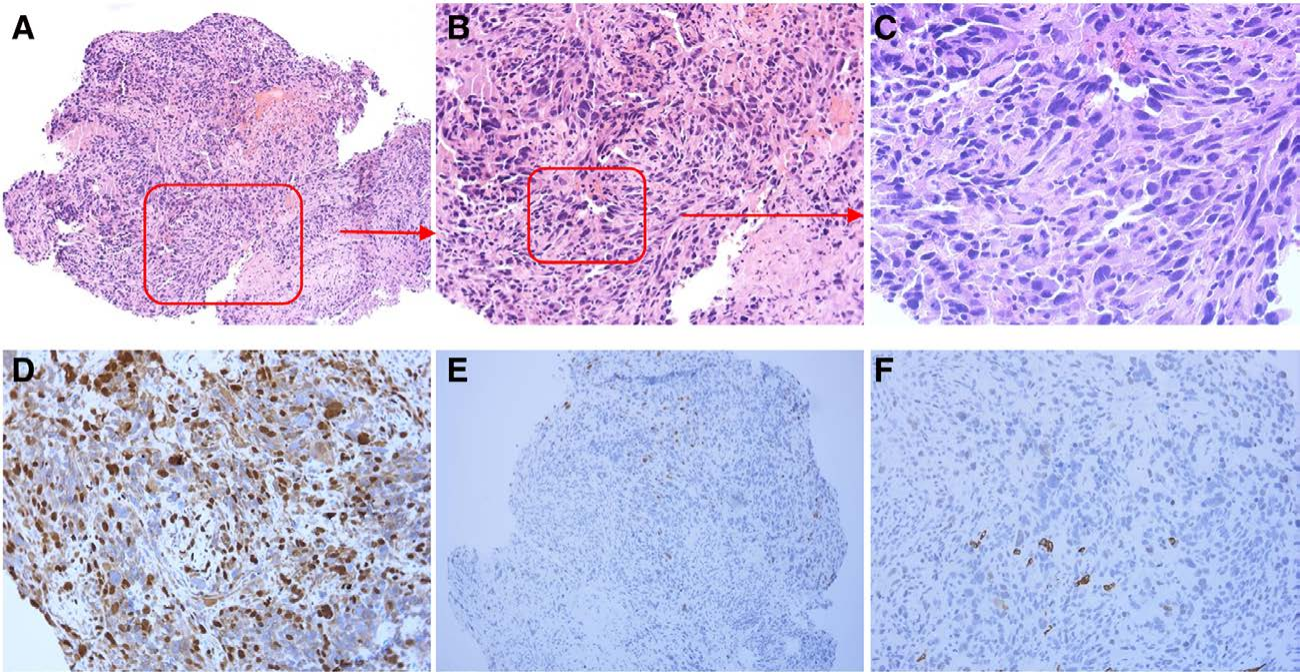
Postoperative pathological images. (A–C) HE staining of the postoperative tissue. Tumor was composed of spindle-shaped, partly polygonal and size-variable cells with an eosinophilic cytoplasm and pleomorphic nuclei. In some areas, cells with bizarrely enlarged nuclei could be detected. Focal necrosis can be seen in the tumor. (100×, 200×, 400× respectively). (D–F) IHC staining of desmin, myogenin, and CK respectively (200×). IHC = immunohistochemistry.

In October 2019, magnetic resonance imaging of the soft tissues on the patient’s back revealed nodules within the postoperative area of the rhabdomyosarcoma. Given this residual tumor, the patient underwent further extended resection combined with clinical recommendations. Postoperative IHC revealed that the tumor was positive for desmin, S100, and EMA and negative for MyoD1, myogenin, CKpan, CD34, SMA, SOX-10, HMB, and MelanA. These findings confirmed the presence of rhabdomyosarcoma (Fig. 1). Surgery was followed by adjuvant chemotherapy using 6 cycles of doxorubicin (40 mg/m^2^) and ifosfamide (2.0 g/m2, q21d) from November 2019 to March 2020. In May 2020, the patient began taking oral anlotinib without any obvious discomfort. During regular review, no obvious abnormalities were observed.

In October 2020, the patient noted the reappearance of a back mass accompanied by pain and gradual mass enlargement. Subsequently, contrast-enhanced chest and abdominal computed tomography (CT) indicated soft tissue density foci on the right side of his back, within the chest wall, and within the right anterior superior abdominal wall, suggesting tumor recurrence. Pulmonary nodules and bone changes were considered to be metastases. In November 2020, after obtaining consent from the patient, his formalin-fixed, paraffin-embedded tumor sample was sent to OrigiMed (Shanghai, China) for next-generation sequencing analysis using the YuanSu IO panel. Molecular testing indicated high MSI-H and high tumor mutational burden (TMB-H, 39.1 Muts/Mb), and IHC showed high PD-L1 expression (CPS, 15). These results suggest that patients may benefit from immunotherapy.

**Figure 2.**
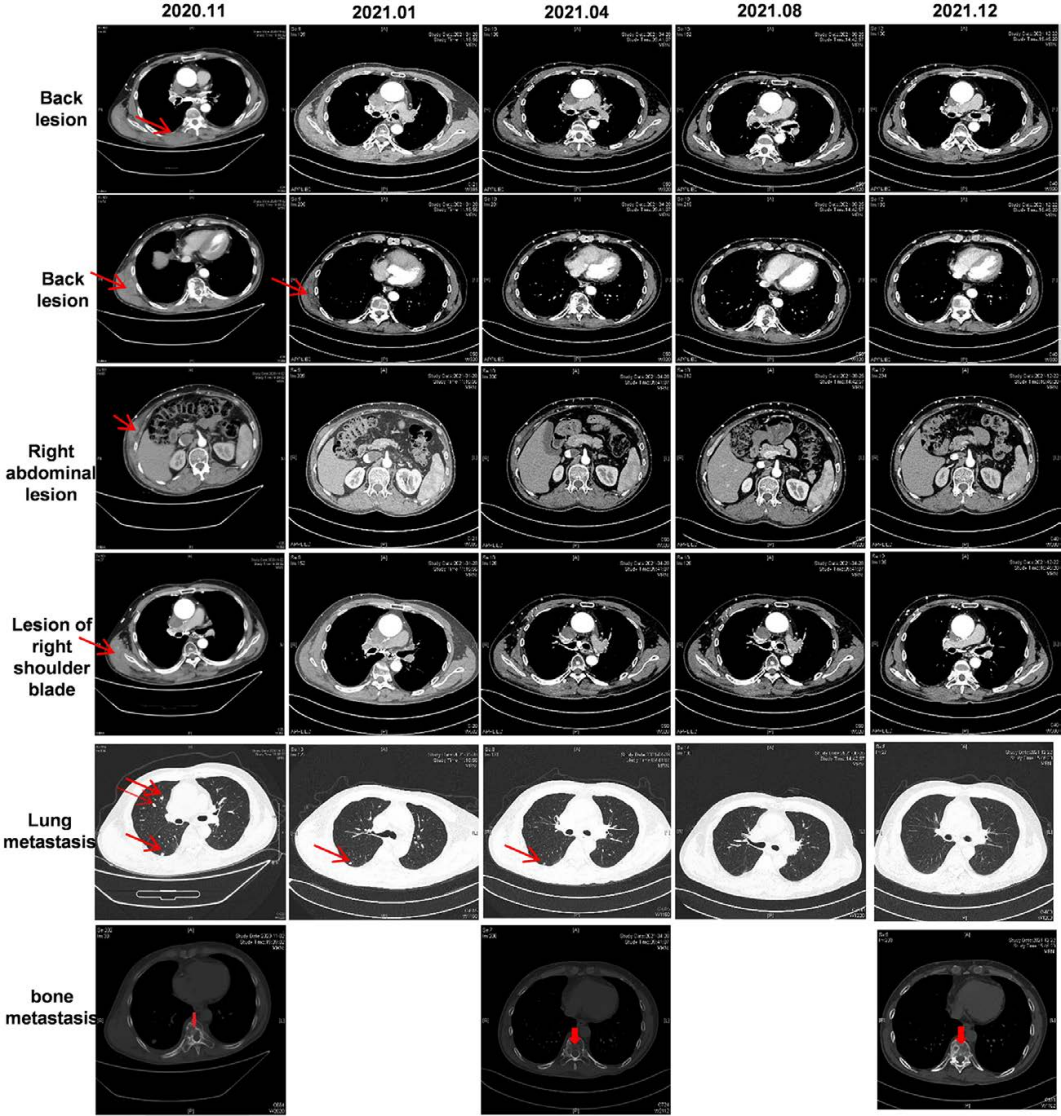
CT images during treatment, including back, lung, and bone metastasis, the red arrow indicates the location of the lesions. CT = computed tomography.

To inhibit bone metastasis, the patient received the programmed cell death protein 1 (PD1) antibody toripalimab (240 mg) and ibandronate (4 mg), beginning in November 2020. The patient has continued anti-rotinib therapy since November 2020. In follow-up restaging performed using a contrast-enhanced chest and abdominal CT scan of the back, abdomen, chest wall, shoulder blade, and both lungs following therapy, the patient displayed a clear response to the therapy, with profound remission for all tumor lesions. Until the date of this report, the patient was still receiving the above-mentioned treatment regimen with good tolerance, and his condition was stable with a partial response (PR) ≥ 17 months (Fig. 2).

## 3. Discussion

RMS can occur as a primary malignancy with a very poor prognosis and can also be a component of a heterogeneous malignancy such as a nonmalignant cell or a teratomatous malignant tumor. The histological distribution of RMS in adults differs from that in young children. RMS can arise at a variety of anatomical sites throughout the body. The most common primary tumor sites include the head and neck region (35%), followed by the genitourinary and extremity primaries.^[4]^ In our case, the RMS was chemoresistant, and next-generation sequencing analysis provided us with resources to determine a novel combination immunotherapy option for our patient.

Over the last decade, multidisciplinary treatment of the disease, based on collaborative group clinical trials, has enabled improvements in chemotherapeutic dosing regimens, local control, and management of treatment-related toxicities. As a result, the 5-year overall survival of children with RMS has exceeded 70%. However, improvements in cure rates have generally been limited to patients with low- and intermediate-risk RMS. To date, no significant progress has been made regarding cure rates for patients with advanced or metastatic RMS.

The current frontline treatment for all risk groups of RMSs is a multimodal approach that comprises chemotherapy, surgical resection and/or radiation therapy, immunotherapy, and new therapeutic approaches. Standard chemotherapy regimens for RMS in North America include vincristine, actinomycin D, and cyclophosphamide (VAC), whereas those in Europe include ifosfamide, vincristine, and actinomycin D (IVA). Patients with high-risk disease require improved treatment. Recent COG studies have employed a more intensive chemotherapy backbone using vincristine, doxorubicin, and cyclophosphamide alternated with ifosfamide and etoposide.^[3,5]^ Since the survival outlook for high-risk patients has historically been poor, a plateau of efficacy may have been reached regarding traditional cytotoxic chemotherapy. Therefore, there is significant interest in the development of targeted, personalized, and effective treatment approaches.

Several comprehensive genomic analyses have identified specific molecular alterations in STS patients. VEGF is one of the main drivers of angiogenesis and plays a crucial role in tumor growth, invasion, and metastasis. In addition to the angiogenic pathway, factors within the proliferative pathway, such as PDGF and c-Kit, are also likely to contribute to the highly malignant phenotype of STS.^[6]^ Taken together, these findings provide a rationale for proangiogenic and proliferative factors to serve as potential targets for the treatment of STS.

Anlotinib is a novel tyrosine kinase inhibitor that selectively targets VEGFR-2, -3, FGFR-1, -2, -3, and-4 with high affinity. In a Phase II study, anlotinib displayed antitumor activity against several STS subtypes that progressed following previous anthracycline-based chemotherapy. The primary endpoint was a progression-free rate at 12 weeks (PFR12 weeks) of 68% and objective response rate (ORR) of 13% (95% confidence interval, 7.6–18%). Median progression-free survival and overall survival were 5.6 and 12 months, respectively. Our patient underwent curative surgery and chemotherapy followed by disease progression after 5 months of sequential monotherapy with anlotinib. Patients with rhabdomyosarcoma of the bone have a poorer prognosis than those with rhabdomyosarcoma at other sites, which may be due to the higher percentage of advanced-stage tumors and technical difficulties associated with local therapy for bone and lung metastatic lesions.

Sarcoma has not traditionally been considered to be a type of immunogenic tumor. However, several studies have indicated that PD-L1 expression can be found in up to 30% to 40% of certain sarcoma subtypes. The presence of infiltrating CD8+ T cells correlated with improved outcomes also suggests that immune response plays a vital role in the natural history of the disease.^[7,8]^ According to several basic and clinical studies, the immune checkpoint axis is a strong therapeutic target for various malignancies. Although the clinical benefits of immune checkpoints as therapeutic targets for soft-tissue sarcomas are unclear, studies have reported the importance of immune checkpoints in sarcomas.

The SARC028 trial was the first prospective multicenter, open-label, phase 2 study for immune checkpoint blockade in patients with advanced soft tissue and bone sarcomas, and pembrolizumab monotherapy has been reported to be associated with clinically meaningful and sustained objective responses in 18% of patients with STS.^[9]^ The Alliance A091401 study evaluated the effectiveness of nivolumab (anti-PD-1) alone or in combination with ipilimumab (anti-CTLA-4) for the treatment of unresectable and metastatic sarcoma. In 38 patients who received nivolumab monotherapy, the ORR was 5%. Responses occur in undifferentiated pleomorphic sarcoma and sarcomas not otherwise specified.

Immunohistochemical evaluation of PD-1/PD-L1 expression within the tumor microenvironment is believed to predict the response to PD-1/PD-L1 inhibitors; however, its predictive value is relatively weak. The mutational burden of tumors and microsatellite instability status are better predictors for the efficacy of immune checkpoint inhibitors, likely because tumors with multiple mutations are more immunogenic and, thus, more responsive to immunotherapy.^[10]^ In the present case, the positive expression of PD-L1, TMB-H, and MSI-H in RMS demonstrated that immunotherapy is a worthwhile treatment option.

Anti-PD-1 antibodies as monotherapy in combination with anti-CTLA-4 antibodies have shown antitumor activity against advanced sarcomas, although the proportion of patients who achieve a response remains modest. Based on the central role of VEGF in maintaining a suppressive immune microenvironment, combination therapies with VEGF blockade and immune checkpoint inhibitors have shown favorable outcomes in advanced sarcomas. In our case, following disease progression with anlotinib monotherapy, the patient achieved a sustained partial response and progression-free survival using a combination of anlotinib and pembrolizumab treatment.

Rhabdomyosarcoma is generally considered to be a “non-immunogenic” tumor. However, our study corroborates that rhabdomyosarcoma is highly variable in biology and that each histologic subtype should be considered as a separate therapeutic challenge, requiring a distinct understanding of immunity and molecular biology. As such, the decision to combine immunotherapy with an anti-VEGF receptor, a tyrosine-kinase inhibitor, remains an individualized decision between a patient and their oncology team. Although it is currently not possible to predict whether patients in similar situations would benefit from combined immunotherapy, our results demonstrate that precision therapies are crucial for optimal individualized management and survival rate elevation.

In conclusion, we reported an RMS patient with TMB-H, MSI-H, and positive PD-L1, who benefited from combined treatment of toripalimab and anlotinib. This is the case of the longest progression-free survival for PD-1 inhibitors on Rhabdomyosarcoma, which supported that positive PD-L1, TMB-H, and MSI-H could be strong beneficial biomarkers for immunotherapy in Rhabdomyosarcoma.

## Acknowledgments

We thank the patient and his family for their support. We thank the colleagues of our hospital who did not sign. We thank our colleagues who have treated this patient.

## Author contributions

**Conceptualization:** Jun Li.

**Data curation:** Xiaogang Sun, Xinyu Wang, Xiaoliang Shi, Jun Li.

**Investigation:** Xiaogang Sun, Xinyu Wang, Jun Zhou, Yali Xu.

**Methodology:** Xinyu Wang, Jun Li.

**Project administration:** Wei Song.

**Resources:** Xiaogang Sun, Xinyu Wang, Yali Xu, Wei Song.

**Software:** Jun Zhou, Hao Zhang, Mian Xu.

**Supervision:** Jun Zhou, Mian Xu.

**Validation:** Jun Zhou, Yali Xu, Hao Zhang, Mian Xu, Jiaojiao Shen, Xiaoliang Shi, Jun Li.

**Visualization:** Hao Zhang, Mian Xu, Wei Song.

**Writing – original draft:** Xiaogang Sun, Xinyu Wang.

**Writing – review & editing:** Xiaogang Sun, Xinyu Wang, Yali Xu, Mian Xu, Jiaojiao Shen, Xiaoliang Shi, Wei Song, Jun Li.
